# Determining the effective dose of esketamine for mitigating pain during propofol injection by Dixon’s up-and-down method: a double-blind, prospective clinical study of drug dose response

**DOI:** 10.1186/s12871-022-01914-z

**Published:** 2022-12-01

**Authors:** Meiyun Tan, Chunyuan Zhang, Wei Zeng, Maofang Chen, Zehui Huang, Ding Huang

**Affiliations:** Department of Anesthesiology, Affiliated Boai Hospital of Zhongshan, Southern Medical University, NO.6 Chenggui Road, East District, Zhongshan, 528400 Guangdong People’s Republic of China

**Keywords:** Esketamine, Propofol injection pain, Effective dose

## Abstract

**Background:**

Propofol is an intravenous (IV) anesthetic medication widely used for procedural sedation, operative anesthesia, and in intensive care unit (ICU), but the incidence of pain during IV infusion can reach 28–90%. Ketamine can attenuate pain associated with IV propofol injection through local and central analgesic effects. Ketamine is gradually being transitioned to its S-enantiomer, esketamine, which has a similar mechanism of action. The purpose of our study is to determine the half effective dose (ED_50_), 95% effective dose (ED_95_), and 99% effective dose (ED_99_) of esketamine for attenuating propofol injection pain using Dixon’s up-and-down method to provide a reference for optimal dose selection for surgeries and procedures.

**Methods:**

Thirty gynecological patients undergoing hysteroscopic surgery were enrolled in a sequential method to determine the effective dose of esticketamine for analgesic propofol injection in order of operation. This study was based on the sequential allocation up-and-down rule designed by Dixon, and each patient was induced by esticketamine combined with propofol. During induction, the target dose of esketamine was first given via venous access in the left hand of the patient, and 30 s later, a fixed dose of 2 mg/kg (1 ml/s) of propofol was given. Patient perception of pain was scored with the verbal rating scale (VRS) every 5 s after the start of the propofol infusion, and the evaluation was stopped once the patient became unresponsive. The dosage of esketamine was increased or decreased up or down according to the patient’s pain response. The initial dose of esketamine was 0.2 mg/kg, and the gradient of adjacent dose was 0.02 mg/kg. If the pain response assessment of the upper patient was positive (+), the dose of esselketamine in the next patient was increased by 0.02 mg/kg; if the pain response assessment of the upper patient was negative (−), the dose of esselketamine in the next patient was decreased by 0.02 mg/kg. The tests were carried out sequentially, with the pain response changing from positive to negative or from negative to positive, and the tests were stopped after at least 6 crossover points, and the effective dose of esticketamine was calculated using probit probability regression analysis.

**Results:**

The ineffective group comprised patients with a positive pain response and the effective group comprised patients with a negative pain response. The 95% CI was set as the confidence interval of effective dose ED value，and we found esketamine’s ED_50_ = 0.143 mg/kg (0.120, 0.162 mg/kg), ED_95_ = 0.176 mg/kg (0.159, 0.320 mg/kg), and ED_99_ = 0.189 mg/kg (0.167, 0.394 mg/kg). The esketamine dose and VRS score during propofol injection were significantly different between the two groups (*P* < 0.05), whereas surgical duration, emergence time, visual analogue scale (VAS) score of postoperative uterine contraction pain, and Riker sedation/anxiety scale (SAS) score were not significantly different. Bradycardia occurred in only one patient during anesthesia induction, while hemodynamics was stable in the rest of the patients without obvious adverse reactions.

**Conclusion:**

Small doses of esketamine combined with propofol can be safely and effectively used for hysteroscopic surgery. We recommended a dose of 0.2 mg/kg IV esketamine before induction of anesthesia to reduce the pain of propofol injection.

**Trial registration:**

Chinese Clinical Trial Registry ChiCTR2100048951. Date of registration: July 19, 2021.

## Background

Propofol is an intravenous (IV) anesthetic medication widely used in for procedural sedation, in operating rooms, and intensive care units (ICUs). Propofol is particularly suitable for outpatient and same-day surgeries because of its strong sedative and hypnotic effects, fast onset, quick recovery, and low incidence of postoperative nausea and vomiting (PONV). However, the incidence of pain from IV propofol injection can reach 28–90% and averages about 60–70% [1, 2]. The burning pain from propofol infusion travels along the blood vessels, causing discomfort and anxiety in patients, and may even result in a traumatic anesthesia experience, making propofol infusion-related pain the major problem encountered in clinical anesthesia practice [3]. More researchers are studying different medications and methods to relieve IV propofol pain, increase comfort during anesthesia, and improve patient satisfaction [4, 5]. Among the many proposed methods of pain control, pre-injection of ketamine is an effective method that reduces propofol injection pain through local and central analgesic effects at a recommended dose of 0.3 mg/kg [6, 7]. Ketamine is a classic N-methyl-D-aspartate (NMDA) receptor antagonist and the only intravenous anesthetic with sedative, analgesic, and anesthetic effects [8]. Ketamine has gradually been replaced by its S-enantiomer esketamine. The two mixtures act in a similar way [9]. Esketamine has a stronger affinity to NMDA receptors, its analgesic and hypnotic intensity is twice that of ketamine, and it is quickly metabolized. Thus, esketamine results in few psychiatric adverse reactions, mild respiratory depression, and a rapid recovery [10–12]. Esketamine has unique pharmacological advantages, but it is not clear whether it is superior to ketamine in relieving the pain of propofol injection. Studies on the effective dose of esketamine in relieving the pain of propofol injection have not been reported.

The purpose of this study was to use Dixon’s up-and-down method [13, 14] to determine the half effective dose, 95% effective dose and 99% effective dose of esticketamine for relieving propofol injection pain, so as to provide reference for clinical medication.

## Methods

### Study design and patient population

Our team was conducting a double-blind, prospective, single-center dose-response clinical study, which was study on effective dose of esketamine, remifentanil and lidocaine in relieving pain of propofol injection in patients of different ages, and the study was a large research project of the effective dose of three drugs (esketamine, remifentanil, and lidocaine) to eliminate the pain of propofol injection in different populations (children, adults, and the elderly). The Ethics Committee of the Affiliated Boai Hospital of Zhongshan (Southern Medical University) gave ethical permission for the study on June 15, 2021 (Ethical Committee No. KY-2021-006-02; Zhongshan, Guang dong, China), and the study (ChiCTR2100048951, 19/07/2021) was been registered in the China Clinical Trials Registry (https://www.chictr. org.cn/abouten.aspx). The study was plan to recruit 200 subjects from different populations according to the actual surgical order. At present, our team had completed the recruitment of 120 subjects, and had completed the dose study of esketamine to eliminate pain in adult propofol injection, which was exactly the experiment reported in this paper. At the same time, our team was currently conducting a dose study of esketamine to relieve pain from propofol injections in children and the elderly, and two other drugs (remifentanil, lidocaine) were also being studied. We were planed to complete all the trials by December 2023. As esketamine was a newly marketed drug in China in recent years, the effect of esketamine on the pain of injection of propofol was not clear, and the adult test was safer than that of children and the elderly. Therefore, our team first choose to conduct the trial of esketamine on the pain of injection of propofol in adults, which was also the reason why we first reported this trial. Thirty gynecological patients who planned to undergo hysteroscopy were included in this trial，and all patients were writted informed consent before participation in this trial. All procedures of this trial followed the tenets of the Helsinki Declaration.

### Inclusion and exclusion criteria

We used the following inclusion criteria: (1) female aged 18–40 years old with a body mass index (BMI) of 18.5–24.9 kg/m^2^; (2) American Anesthesiologists Association (ASA) Physical Status I or II and Mallampati grade I or II; (3) no contraindications for esketamine, opioids, or propofol; (4) no history of drug abuse; and (5) no systemic neurological, cardiac, pulmonary, hepatic, or renal disease.

We used the following exclusion criteria: (1) difficult airway; (2) patients with hypertension, hyperthyroidism, myasthenia gravis, schizophrenia, or epilepsy; (3) severe cardiopulmonary or cerebrovascular diseases; (4) patients who had recently taken psychotropic medications or analgesics; and (5) those who were allergic or addicted to opioids or esketamine.

### Preoperative preparation and anesthesia protocol

Dixon’s up-and-down method was a classical method to determine the effective dose of drugs [13, 14]. Usually, an initial dose was set, and the dose used by the next patient was increased or decreased according to the response of the previous patient. Usually, the patient had a reaction to no reaction, or no reaction to reaction, which was a crossing point. At least 6 crossover points were required, and the sample size was about 20–40, which can meet the criteria for termination of the experiment [13, 14]. We selected 30 patients who underwent gynecological hysteroscopic surgery under general anesthesia. All patients fasted for 6 h pre-procedure and had imbibed no water for 2 h. Venous access was placed in the left hand of all patients 30 min pre-operatively, and 6–8 ml/min of lactated Ringer’s solution was infused. After entering the surgical suite, all patients received oxygen at 2 L/min by nasal cannula and the electrocardiogram (ECG), heart rate (HR), mean arterial blood pressure (MAP), and pulse oximetry (SpO_2_) were monitored. Patients rested for 5 min after entering the operating room. IV anesthesia was induced and the target dose of esketamine was given first, followed by a fixed dose of propofol at 2.0 mg/kg 30 s later, infused at 1 ml/s using a syringe pump. After the propofol solution was given, the patient’s pain response was assessed every 5 s with the verbal rating scale (VRS) [7] and the degree of sedation was observed. The pain assessments were stopped once the patient became unresponsive.

The esketamine dose was titrated across study participants based on Dixon’s up-and-down method. The initial target dose was 0.2 mg/kg and the next sequential dose was adjusted up or down by 0.02 mg/kg. If the pain response assessment of the previous patient was positive (+), the dose of esketamine in the next patient was increased by 0.02 mg/kg. If the pain response assessment in the previous patient was negative (−), the dose of esketamine in the next patient was reduced by 0.02 mg/kg, and the tests were carried out sequentially. Patient pain response ranged from positive to negative or from negative to positive, and the test was stopped after at least six intersections [13, 14]. Pain response was assessed according to the VRS [7] as follows: (1) *Painless* – when asked, the patient reported no pain, score = 0; (2) *Mild pain* – when asked, the patient reported pain and no painful expressions or movements were observed, score = 1; (3) *Moderate pain* – the patient reported pain voluntarily, or when asked, and there were movements such as withdrawing hands, score = 2; and (4) *Severe pain* – the patient reacted strongly and there were movements such as frowning, withdrawing hands, and crying, score = 3. For our study, patients were divided into two groups (effective or ineffective) based on their reaction to esketamine as measured with the VRS score. We designated 0 points as a negative pain response (−) for the effective group and 1–3 points on the VRS as a positive pain response (+) for the ineffective group.

Atropine 0.01 mg/kg iv if bradycardia is present, and ephedrine 0.1 mg/kg iv if hypotension is present, repeated as necessary. Esketamine solution was diluted with normal saline to control the total volume to 10 mL, which was prepared by a dispenser who did not know the test protocol before induction of anesthesia. An anesthesiologist who did not know the dose of the esketamine solution performed propofol IV. And another anesthesiologist judged the pain response, and reported the pain reactions to the dispenser, so as to obtain the next patient esketamine solution. Our study was double-blind in order to reduce the interference of human factors and obtain the effective dose of esketamine more accurately.

### Observation indicator

Baseline blood pressure and HR were defined as the average of two measurements spaced within 5 min (T0) before the onset of anesthesia. The changes of HR, MAP, and SpO_2_ in the effective and ineffective groups were recorded at the following time points: 5 min after entering the room (T0), 1 min before propofol administration (T1), 1 min after propofol administration (T2), 3 min after the administration of propofol (T3), and 5 min after recovery (T4). The mean esketamine dose, VRS score of propofol injection pain, surgical duration, emergence time, visual analog scale (VAS) score of postoperative uterine contraction pain [15], and Riker sedation/anxiety scale (SAS) score [16] were recorded. The occurrence of adverse reactions was recorded, including allergic reaction, hypotension, bradycardia, respiratory depression (SpO_2_ < 90%), delayed emergence, nausea and vomiting, and postoperative agitation.

## Statistical analysis

According to previous sequential method studies, the sample size usually requires 20–40 patients [13, 14]. In our preliminary trials, we found that the sample size for completing a crossover point was about three to four cases. Therefore, we estimate that the sample size for completing the six crossover point was between 18 and 24 cases. Considering an attrition rate of 10%, we included 30 patients in our trial. The ED of esketamine’s was determined by Dixon’s up-and-down method [7, 14], and dose-response data were analyzed by Probit regression [7]. Data were presented as mean and 95% confidence interval [mean (1.96SD), 95% CI]. Dose values were entered as x value, Y was the response as a percentage. The regression coefficients was obtained by regression analysis, and the ED values were obtained from interpolation of the linear probit regression plot, and generation of the esketamine’s dose-response plot was obtained secondarily. Shapiro-Wilk was used to test the normal distribution in SPSS25.0 statistical software, *P* > 0.05 indicates that the data conform to normal distribution. Student’s t test was used for normal distribution data as mean ± standard deviation (^−^*x ± s*). And there were no non-normal variables in the chart data listed in this paper. We assume that the *P* value is two-sided, and *P* < 0.05 was considered statistically significant.

## Results

**Fig. 1:**
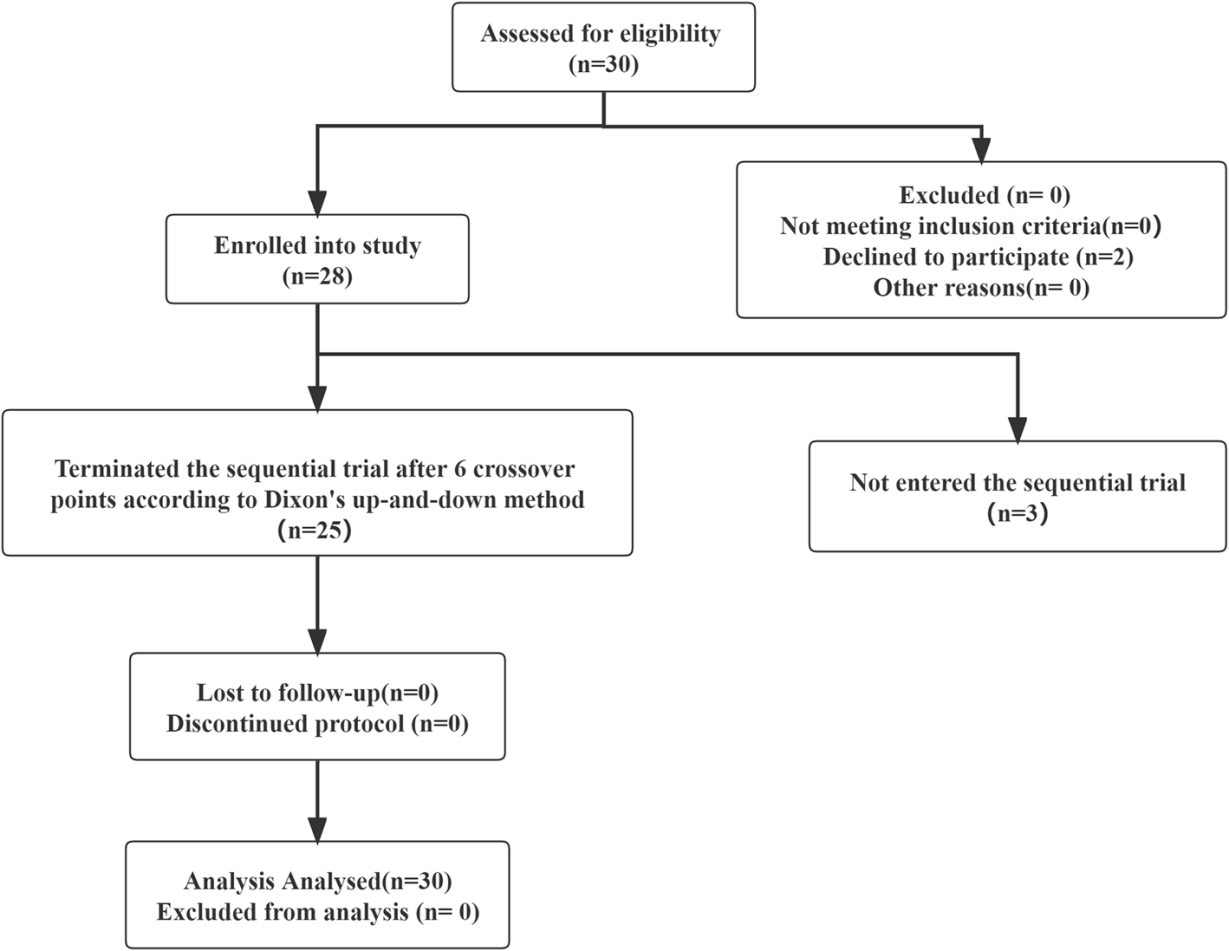
The timeline of the wholes study

The sequential trial was carried out according to the operation sequence of patients，The timeline of the wholes study was shown as Fig. 1. Two patients who withdrew from the trial after entering the operation room were excluded, and three patients did not enter the trial. After the 25th patient, six crossover points appeared and the trial was terminated. In the end, there were 14 patients in the effective group (negative pain response) and 11 patients in the ineffective group (positive pain response). Patient demographic data and characteristics in the effective and ineffective groups are shown in Table 1, and there was no significant difference in age, weight, BMI, and ASA grade between groups (*P* > 0.05).

**Table 1:** Demographic data and patient characteristics

Group	Number of cases	Age (yr)	Weight (kg)	BMI (kg/m^2^)	ASA (I/II cases)
Effective	14	36.4 ± 9.0	57.3 ± 5.8	22.1 ± 1.6	10/4
Ineffective	11	34.5 ± 6.7	56.7 ± 5.7	22.5 ± 1.0	8/3

Values are expressed as mean ± SD or number of patients; *BMI* Body mass index, *ASA* American Society of Anesthesiologists physical status

There was no significant difference in HR, MAP, and SpO_2_ between the two groups between the T0–T4 time points (*P* > 0.05). There was no significant difference in surgical duration, emergence time, VAS score of postoperative uterine contraction pain, or SAS score between the two groups, but there were significant differences in esketamine dose and VRS score of propofol injection pain between the groups (*P* < 0.05) (Table 2).

**Table 2:** Comparison of the experimental conditions of the two groups of patients

Group	Number of cases (n)	Esketamine (mg/kg)	Surgical duration (min)	Awakening time (min)	VRS score	VAS score	SAS score
Effective	14	0.16 ± 0.2	10 ± 1.1	8.7 ± 1.1	0 ± 0	1.1 ± 0.4	3.8 ± 0.4
Ineffective	11	0.14 ± 0.2^a^	9.4 ± 1.2	8.4 ± 1.2	1.3 ± 0.5^a^	1.4 ± 0.5	3.9 ± 0.3

Values are expressed as mean ± SD or number of patients. Compared with the effective group, ^a^ *P* < 0.05

**Fig. 2:**
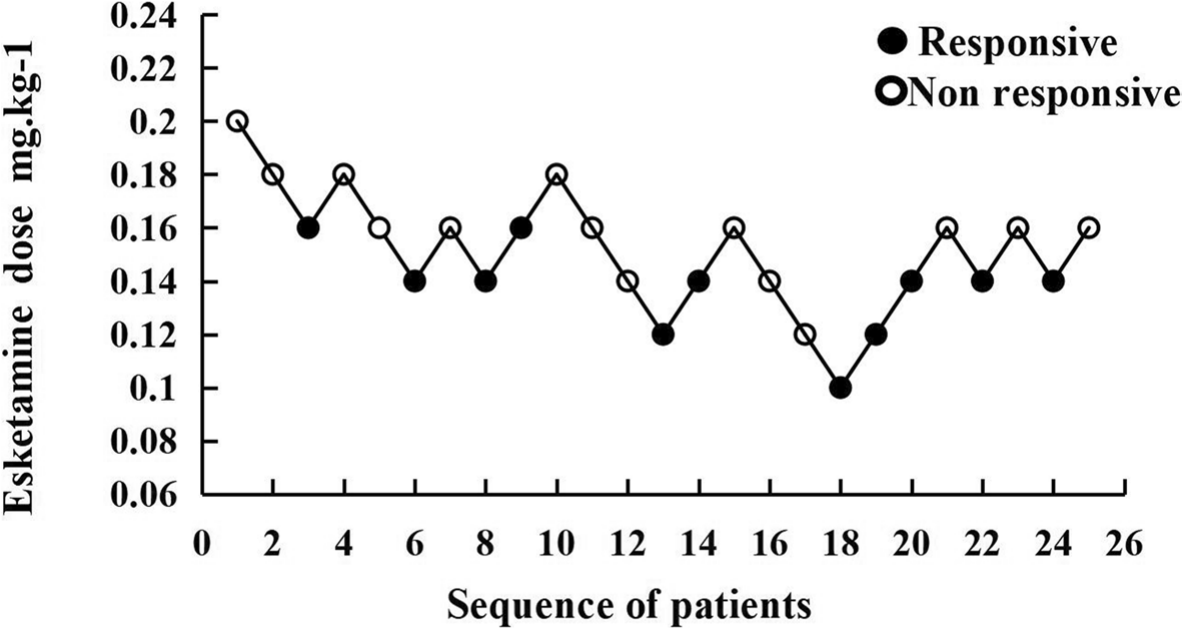
The sequential trial of esketamine for attenuation of propofol injection pain

**Fig. 3:**
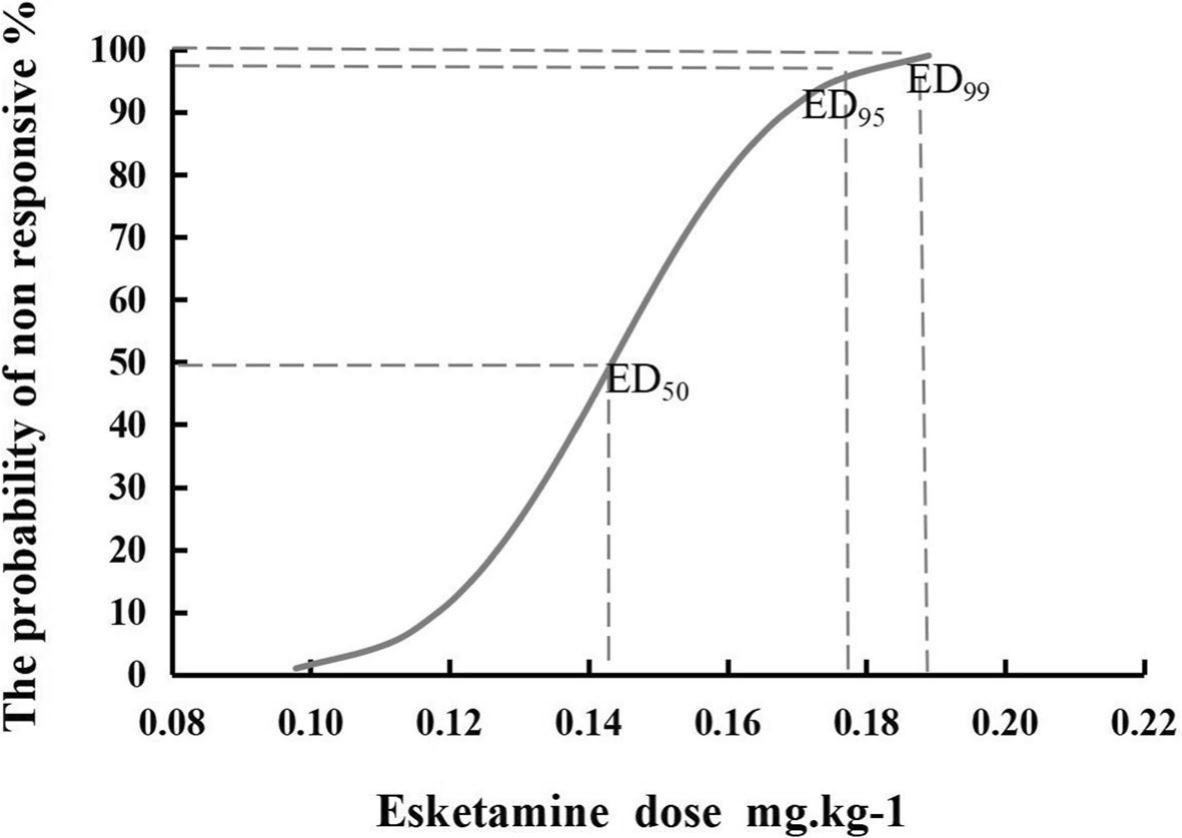
The dose–response curve of esketamine for attenuation of propofol injection pain

The 95% CI was set as the confidence interval of effective dose ED value, according to the sequential analysis, the probit method was used to obtain esketamine’s ED_50_ as 0.143 (0.120, 0.162) mg/kg, ED_95_ as 0.176 (0.159, 0.320) mg/kg, and ED_99_ as 0.189 (0.167, 0.394) mg/kg. Our results showed esketamine at 0.2 mg/kg to be > 99% effective in reducing pain from propofol injection. The sequential test of esketamine to attenuate propofol injection pain and the dose-response relationship was shown in Figs. 2 and 3.

During the trial, only one patient in the effective group had an adverse cardiovascular reaction, while the other patients did not have hypotension, hypertension, tachycardia, etc. There was no incidence of other types of adverse reactions such as allergic reactions, nausea, vomiting, postoperative agitation, or hallucinations.

## Discussion

The ED_50_, ED_95_, and ED_99_ of esketamine for mitigating propofol injection pain were 0.143 (0.120, 0.162) mg/kg, 0.176 (0.159, 0.320) mg/kg, and 0.189 (0.167, 0.394) mg/kg, respectively, identified using a sequential method. The mechanisms behind propofol injection pain are still unclear. It is currently believed that high concentrations of free propofol can induce pain through direct stimulation and activation of the kallikrein-kinin system, activation of nociceptive cis-receptor potential ion channels, and changes in osmotic pressure [4]. Prophylactic methods such as IV pre-injection of lidocaine, opioids, ketamine, benzodiazepines, metoclopramide, flurbiprofen axetil, cooling, heating or diluting the propofol liquid, or choosing a larger vein for cannulation can reduce the incidence and intensity of propofol injection pain to varying degrees by blocking the pain-inducing mechanism of propofol [4, 5].

Intravenous lidocaine, which has the dual effects of local anesthesia and central analgesia, is commonly used at a dose of 0.5–1 mg/kg to reduce the pain of propofol injection. Nonetheless, there is a failure rate of 13–32%. Continuing to increase the lidocaine dose does not improve effectiveness but instead increases the potential risk of local anesthetic toxicity [7, 17]. Iwata et al. [18] found that ketamine, which also has the dual-action mechanism of promoting local and central analgesic effects, completely eliminated propofol injection pain after high-dose (1 mg/kg) pretreatment, but was prone to inducing sympathomimetic effects, hallucinations, and other adverse mental reactions, which limits its clinical applications. Saadawi et al. [19] confirmed low-dose ketamine pretreatment at 0.4 mg/kg is effective in reducing propofol injection pain. This analgesic effect is superior to lidocaine and pethidine. The use of the proper ketamine dose for this indication is important [20, 21]. Large-dose (≥ 1 mg/kg) IV injection produces a general anesthetic effect, while small-dose (< 1 mg/kg) IV injection is mainly used for analgesia and local anesthesia The intensity of ketamine’s sympathomimetic effect and the probability of adverse reactions such as agitation and hallucinations are also dose-dependent. Small doses of ketamine have a weak sympathomimetic effect and few adverse reactions, but the effect of relieving propofol injection pain is not satisfactory [20, 21].

Esketamine is a dextrorotatory isomer derived from ketamine through separation and purification. Its mechanism of action and pharmacological characteristics are similar to ketamine, and it primarily exerts anesthesia and analgesia by acting on both NMDA and opioid receptors [9]. Esketamine has a stronger affinity for NMDA receptors than ketamine and is twice as potent [9–11]. Esketamine has a prominent dosing advantage. Small doses of esketamine have weak sympathomimetic effects and mild circulatory inhibition. This results in stable hemodynamics and a low incidence of adverse reactions [22, 23]. Esketamine has a rapid onset of action, higher clearance rate, and faster recovery time. Therefore, esketamine has faster and more potent analgesic effects with fewer adverse reactions than ketamine [10, 11].

Presently, esketamine is increasingly replacing ketamine in clinical practice in China, especially in the application of short-duration and same-day surgeries [14, 20, 21, 24]. Our research focused on determining the effective dose of esketamine to relieve propofol injection pain, and observing the clinical effect of low-dose esketamine in reduce pain from propofol injection.

We determined esketamine’s effective doses for mitigating propofol injection pain include an ED_50_ of 0.143 (0.120, 0.162) mg/kg, ED_95_ of 0.176 (0.159, 0.320) mg/kg, and an ED_99_ of 0.189 (0.167, 0.394) mg/kg. The ED_50_ and ED_95_ of esketamine were significantly lower than the ED_50_ (0.227) and ED_95_ (0.283) of ketamine determined by Wang et al [7] Our results show esketamine at 0.2 mg/kg is > 99% effective in reducing pain from propofol injection, but Wang et al. [7] showed that the probability of mitigating propofol injection pain at this dose of ketamine is < 50%. Our results thus further support that esketamine has a higher potency [10, 11]. Since the potency of esketamine is twice that of traditional ketamine, esketamine is often used at 1/2 the dose of ketamine in clinical empirical dosing. However, our test results found the ED_50_ (0.143) and ED_95_ (0.176) doses of esketamine for reduction of propofol injection pain were not 1/2 of the doses of ketamine ED_50_ (0.227) and ED_95_ (0.283) determined by Wang et al [7] Our findings also provide a better reference for clinical empirical dosing and support a dose of 0.2 mg/kg of esketamine for attenuating propofol injection pain, which is smaller than the low-dose esketamine (0.3–0.5 mg/kg) combined with propofol in previous studies [11, 12, 15]^.^ Nonetheless, the smaller doses of esketamine used in our study resulted in a good analgesic effect and fewer side effects; however, our findings must be further verified in broad clinical use amongst a more heterogenous population of patients.

During this trial, only one patient developed hypotension after propofol induction and no other adverse reactions occurred. There were no significant differences in HR, MAP, and SpO_2_ between the two groups of patients before and after induction with propofol. Notably, after intravenous injection of esketamine (1 min before propofol administration), neither HR nor MAP was significantly increased. This may be because the sympathomimetic effect of low-dose esketamine is weak. Neither HR nor MAP was significantly decreased at 1 and 3 min after propofol induction. This may be due to the weaker sympathomimetic effect of esketamine at low doses, which counteract the inhibitory effect of propofol on the cardiovascular system [7, 24]. Before and after propofol induction, SpO_2_ was stable and no respiratory depression was observed, which may be related to the respiratory stimulatory effect of low-dose esketamine, which alleviated the inhibitory effect of propofol on respiration [7, 15]. For ethical reasons, we did not set a propofol test group. Instead, we referred to the results of previous studies on propofol injection alone [1, 2].

Our study had the following limitations: (1) Our study population was entirely female, therefore differences in response to 0.2 mg/kg esketamine between sexes were not evaluated; (2) We did not use an anesthesia depth monitor. It was more clinically meaningful to use the bispectral index (BIS) or Narcotrend to monitor the changes in the degree of sedation and the loss of consciousness during the induction process; (3) No ketamine control group was used. Since ketamine has been essentially withdrawn from our local market and was therefore unavailable, we used previous research results with ketamine as a control. The comparison between the ketamine and esketamine had certain limitations. However, studies have shown low dose esketamine was safe and effective and had a favorable side effect profile; and (4) The sequential method [13] was a classic method for determining the effective dose of medications. This method was efficient and reliable and can be used in studies with a small sample size. This study was a clinical pharmacological trial estimated the efective doses of esketamine to relieve pain associated with propofol injection, was not a randomized controlled trial. This is a single-center study, and the surgical type is hysteroscopy, which is relatively single and limited. Thus, the results of this study should be further confrmed by the large-scale, multi-center, randomized, controlled trials.

## Conclusion

In our trial of female patients undergoing hysteroscopic surgery, the ED_50_ of 0.143 (0.120, 0.162) mg/kg, ED_95_ of 0.176 (0.159, 0.320) mg/kg, and ED_99_ of 0.189 (0.167, 0.394) mg/kg of esketamine were measured using the Dixon’s up-and-down method with no serious adverse events. According to the dose-effect curve, we recommend clinical use of 0.2 mg/kg IV esketamine, which is safe and effective as a pre-propofol injection to relieve pain associated with propofol injection.

## Data Availability

The datasets analysed during the current study are available from the cor-responding author on reasonable request.

## Abbreviations

IV: Intravenous
ICU: Intensive Care Units
ED50: Half Effective dose
ED95: 95% Effective Dose
ED99: 99% Effective Dose
VRS: Verbal Rating Scale
PONV: Postoperative Nausea and Vomiting
NMDA: N-methyl-D-aspartate
ECG: Electrocardiogram
HR: Heart Rate
MAP: Mean arterial blood pressure
SpO_2_: Pulse Oximetry
SAS: Riker Sedation/AnxietyScale
PACU: Postanaesthesia care unit
